# Paclitaxel-Induced Palmar-Plantar Erythrodysesthesia

**DOI:** 10.7759/cureus.11507

**Published:** 2020-11-16

**Authors:** Ahsan Wahab, Maria Khakwani, Hamid Ehsan, Naresh Bellam

**Affiliations:** 1 Internal Medicine, Baptist Medical Center South, Montgomery, USA; 2 Internal Medicine, Lahore Medical and Dental College, Lahore, PAK; 3 Internal Medicine, MedStar Union Memorial Hospital, Baltimore, USA; 4 Hematology and Oncology, Prattville Campus, Montgomery Cancer Center, Prattville, USA

**Keywords:** paclitaxel, palmar-plantar erythrodysesthesia, hand-foot syndrome

## Abstract

Palmar-plantar erythrodysesthesia (PPE) is an uncommon adverse event with paclitaxel. We report a case of PPE due to paclitaxel to create awareness and review management strategies. A 61-year-old female with locally advanced lobular breast cancer was started on neoadjuvant chemotherapy with four cycles of dose-dense doxorubicin and cyclophosphamide. She completed these chemotherapy cycles uneventfully and was started on weekly paclitaxel (80mg/m^2^) with a gap of two weeks. After receiving the sixth dose of paclitaxel, the patient presented with erythema, swelling, and discomfort of her hands and feet, interfering with her quality of life due to difficulty in carrying out daily routine activities. The changes were acute, occurred within a few days after the sixth dose of paclitaxel, and were consistent with PPE grade 2. Paclitaxel was discontinued, and the patient was switched to docetaxel every three weeks for two cycles. She used emollients and moisturizing creams for her local symptoms, after stopping paclitaxel, erythema, swelling, and discomfort of her hands and feet resolved within two weeks. She did not have a recurrence of these symptoms with docetaxel. Paclitaxel can cause PPE. Its incidence in the literature might be underreported. Discontinuation of paclitaxel can reverse skin toxicity and improve patient’s quality of life.

## Introduction

Taxanes (paclitaxel, docetaxel, cabazitaxel), anti-neoplastic medicines, are cytotoxic drugs that impair the microtubular function and interfere with cellular mitosis. Paclitaxel (Taxol), one of the taxanes, is used for ovarian cancer, breast cancer, and non-small cell lung cancer in addition to other malignancies. It causes myelosuppression, neuropathy, fatigue, skin/mucosal, and nail toxicity [1]. Acral erythema or palmar-plantar erythrodysesthesia (PPE), also known as hand-foot syndrome, is not a commonly reported adverse effect of paclitaxel with an occurrence rate of 1.5% to 3.2% reported in the literature [2-4]. Common drugs causing PPE are cytarabine, doxorubicin, 5-fluorouracil, capecitabine, and docetaxel [5,6].

PPE syndrome presents with initial symptoms of palmoplantar dysesthesia followed by symmetrical erythema and swelling of palms and soles, which can also extend to the dorsal and lateral surfaces of hands and feet. Vesicles, bullae, blisters, and desquamation may also ensue [7]. If severe, PPE interferes with the instrumental and self-care activities of daily life and may cause a poor quality of life. We report a patient with PPE due to paclitaxel to create awareness and review management strategies. 

## Case presentation

A 61-year-old female with a history of diabetes mellitus, hypertension, and dyslipidemia noticed a swelling on the medial aspect of her right breast, which resolved after a week. On examination, she had a 4 cm lesion in the tail of the right breast in addition to palpable axillary lymph nodes. She also reported a fullness in her right breast, and a mammogram was performed. The mammogram revealed a large mass in the right axillary tail along with enlarged axillary lymph nodes, strongly suggestive of malignancy. Breast biopsy showed a multifocal invasive lobular breast cancer. Axillary lymph node biopsy also confirmed malignancy. Cancer cells were strongly estrogen-receptor (ER) positive. Human epidermal growth factor receptor-2 (HER2) and progesterone-receptor (PR) were negative. Staging workup with a computed tomography scan of the chest, abdomen, and pelvis and bone scan was negative for metastatic disease. She was started on four cycles of dose-dense neoadjuvant AC (doxorubicin, 60mg/m^2^ for two weeks; cyclophosphamide, 600mg/m^2^ for two weeks) chemotherapy. Pegfilgrastim (granulocyte colony-stimulating factor) was used after each AC cycle. Two weeks after the completion of AC, the patient was started on a weekly paclitaxel dose (80mg/m^2^) for 12 weeks. Pegfilgrastim was not used with paclitaxel. After receiving the sixth dose of weekly paclitaxel, she called in complaining about significant erythema, swelling, and discomfort of her hands and feet. During this period, home medications (metformin, atorvastatin, clonidine, hydrochlorothiazide, and lisinopril) remained unchanged, introducing no significant drug interactions or reactions. Her presentation was consistent with PPE, grade 2 based on National Cancer Institute (NCI) Common Terminology Criteria for Adverse Events (CTCAE) [8]. 

**Figure 1:**
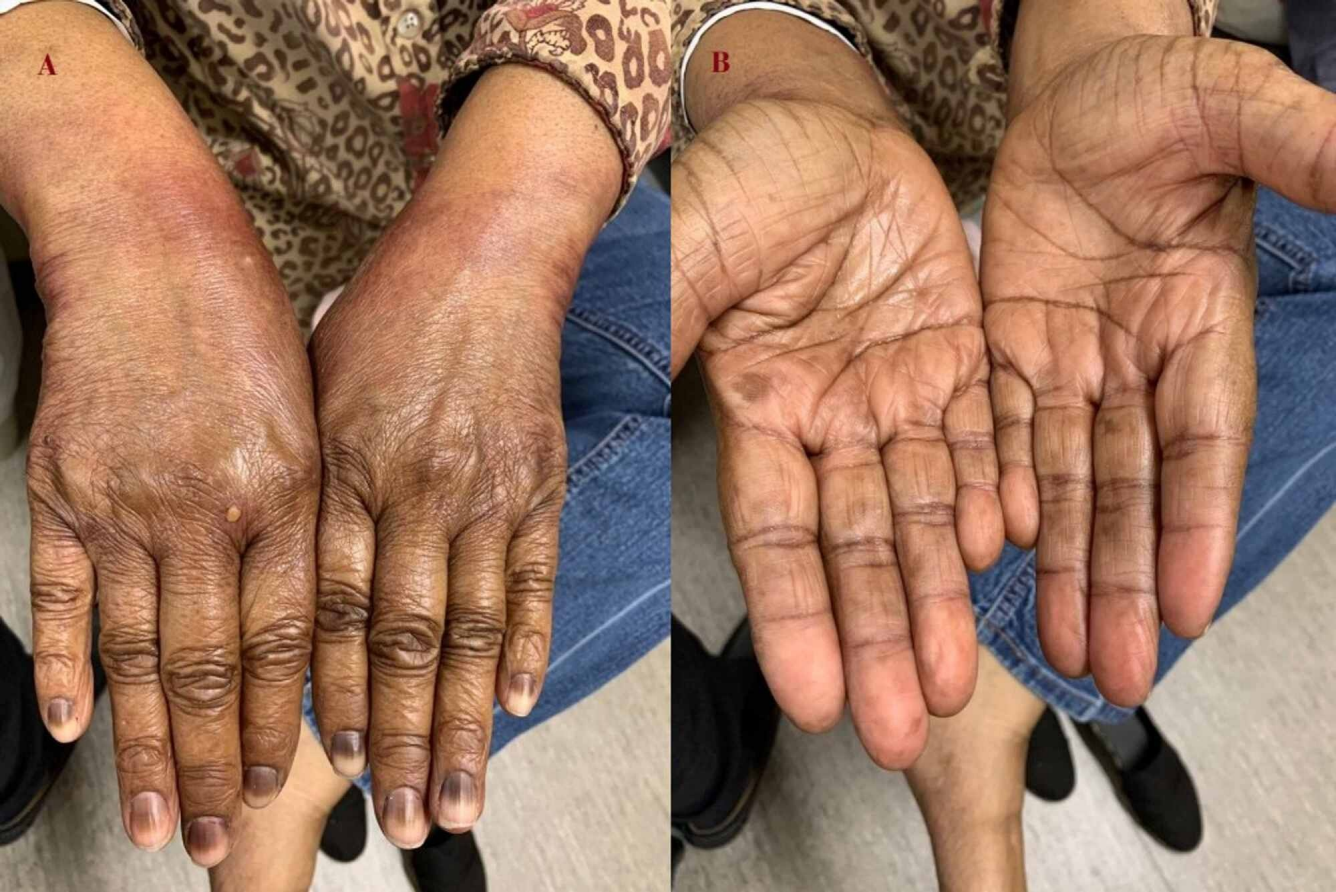
Paclitaxel-induced palmar-plantar erythrodysesthesia A) dorsal surface of hands showing prominent erythema, swelling and hyperpigmentation, B) palmar surface of hands with mild erythema and pigmentary changes

**Figure 2:**
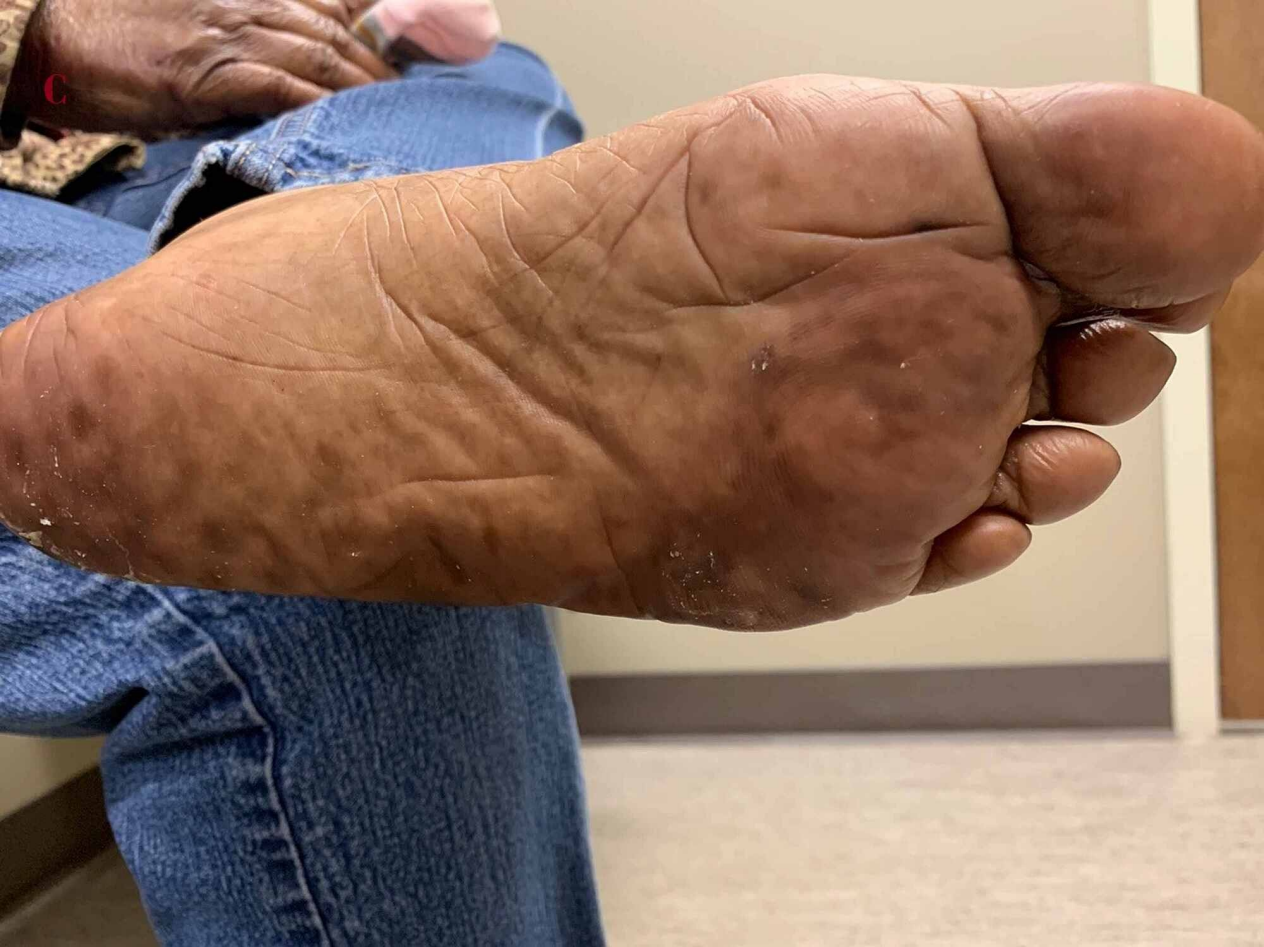
Plantar surface of the left foot showing erythema, pigmentation, and desquamation of the skin, consistent with palmar-plantar erythrodysesthesia

On examination, she was noted to have prominent erythema of her hands and feet (Figure 1). Erythema was more pronounced on the dorsum of her hands (Figure 1A) but was seen on the palmar surface as well (Figure 1B). She had horizontal deep brown pigmentary changes of all of her fingernails, consistent with transverse melanonychia. Feet had mild erythema with some desquamation (Figure 2) 

These skin changes were concerning for the patient and interfered with her quality of life. Though paclitaxel could have been continued safely with the supportive management, it was discontinued based on the patient’s request, and the patient was switched to docetaxel every three weeks for two cycles. As such skin toxicity is common with the docetaxel [9,10], especially with a weekly schedule compared with a three-weekly schedule, we used a three-weekly schedule of docetaxel to avoid the recurrence of symptoms [11]. The patient was counselled that symptoms of PPE could recur with docetaxel as well. Meanwhile, she continued to use emollients and moisturizing creams for her local symptoms, after stopping paclitaxel, erythema, pain and swelling resolved within two weeks. She did not have a recurrence of these symptoms while getting docetaxel and subsequently complained of mild neuropathy type symptoms in the upper extremities. We considered other potential causes of PPE in our patient. Though both cyclophosphamide and doxorubicin can cause such skin toxicity and combination therapies are often implicated in such cases [5,6,12], paclitaxel was considered the most likely etiology of PPE in our patient. First, PPE occurred as an acute reaction within a few days after the sixth dose of paclitaxel. Second, PPE developed eight weeks after the completion of AC, and therefore, it was highly unlikely that the etiology of PPE was AC. Third, though pegfilgrastim was reported to predispose to PPE in the literature [12], it was not implicated in our case as we did not use it with paclitaxel. Fourth, PPE resolved when paclitaxel was discontinued and did not recur in our patient. We calculated the Naranjo scale score to estimate the probability of PPE due to paclitaxel [13]. Naranjo scale score for PPE was six, representing a high probability of its occurrence due to paclitaxel. Post-chemotherapy, she was referred to a surgical team for surgical resection of the tumour.

## Discussion

Compared to other chemotherapy drugs, taxanes, especially paclitaxel, are an uncommon cause of PPE, seen in about 5-10% of treated patients [4]. In contrast to the prominent involvement of palms and soles due to other agents, taxane-induced PPE involves the dorsal surface of hands more distinctly [10,14,15]. PPE occurs due to the cytotoxicity of basal keratinocytes and epidermis [4,6]. Histologically, there is vacuolar degeneration of the basal layer of epidermis with sparse necrotic keratinocytes in mild cases. This may extend to full-thickness epidermal necrosis in severe cases [5]. A predilection for hands and feet may be due to 1) local trauma, 2) photo exposure, 3) a rich capillary network preferentially infusing a higher concentration of chemotherapy drugs, 4) quicker turnover of keratinocytes, and 5) greater density of eccrine glands [4,6,16]. There are only six reports of paclitaxel-induced PPE mentioned in the literature [2,3,17-20].

De Argila et al. were the ones who first reported PPE in 1996 due to 3-weekly paclitaxel in a female patient [17]. Acral changes in their patient occurred after the fourth cycle of paclitaxel [17]. Merimsky and Inbar reported PPE’s first case in 2000 due to weekly paclitaxel (80 mg/m2) in combination with trastuzumab. This patient developed acral erythema after the seventh dose of paclitaxel. Discontinuation of therapy led to the resolution of PPE [18].

Assi et al. described another female presenting with macular erythema, edema, and dysesthesia in sun-exposed areas involving face, neck, hands, and feet [3]. Acral erythema occurred after the sixth dose of paclitaxel (80mg/m^2^), and the remaining treatments were continued despite toxicity. Erythema progressed to ulcerations and blisters and later resolved with mild residual pigmentation. Kataria et al. reported a male patient with PPE due to paclitaxel, who presented with erythema and desquamation of hands and feet after a single cycle of paclitaxel (175 mg/m2) and carboplatin, with consequent interruption of the chemotherapy [2]. Administration of paclitaxel (135mg/m^2^) in one patient caused severe oral mucositis and acral erythema, progressing to erythema multiforme major. Such severe toxicity was thought to be precipitated by significant hepatic dysfunction in this patient [19]. Cruz et al. reported another patient with mild palmar-plantar erythrodysesthesia after the second dose of weekly paclitaxel and worsened more on each subsequent cycle until chemotherapy was completed [20]. Previously reported cases of paclitaxel-induced PPE are summarized in table 1.

**Table 1:** Case Reports of Palmar-Plantar Erythrodysesthesia Associated with Paclitaxel F: Female, M: Male, PPE: Palmar-plantar erythrodysesthesia, yo: Year old. *Our case report.

Author, Year	Demographics of patient	Type of cancer	Time-lapse between paclitaxel start and occurrence of PPE	Dose of paclitaxel	Management strategy
De Argila et al.,1996 [17]	42-yo-F	Breast cancer	Two days after 4^th^ cycle (~82 days)	310-400 mg q20 days	Oral pyridoxine. Completed 10 cycles of paclitaxel until brain metastasis and then stopped. Resolution within 2 months.
Payne et al., 1996 [19]	60-yo-F	Breast cancer	8 days	135 mg/m^2^ (single dose)	Refused further chemotherapy.
Merimsky et al., 2000 [18]	42-yo-F	Breast cancer	After 7^th^ cycle (~49 days)	80 mg/m^2 ^weekly	Discontinuation of therapy.
Cruz et al., 2010 [20]	77-yo-F	Breast cancer	After 2^nd^ cycle (~14 days)	Weekly dose	Resolution after completion of chemotherapy.
Assi et al., 2013 [3]	72-yo-F	Breast cancer	After 6^th^ cycle (~42 days)	80 mg/m^2^ weekly	Avoidance of sun exposure and use of sunscreens/emollients. Paclitaxel was continued despite PPE.
Kataria et al., 2018 [2]	52-yo-M	Oral cancer	13 days	175 mg/m^2^ (single dose)	Supportive care and change in chemotherapy. Oral pyridoxine.
Wahab et al., 2020*	61-yo-F	Breast cancer	After 6^th^ cycle (~42 days)	80 mg/m^2^ weekly	Switched to docetaxel with the resolution of symptoms. No recurrence of PPE.

Our case is unique as it describes palmar-plantar erythrodysesthesia due to weekly paclitaxel, with only six reported cases in the literature to the best of our knowledge (Table 1). Such findings are of particular concern in a female patient due to their potential to cause significant psychological distress in addition to interfering with the quality of life and daily routine activities. Significant PPE and inflammatory changes of hands may cause impaired movements at the small joints given pain and distress and cause functional limitations while performing the fine manual work compromising accuracy and dexterity. Skin and nail changes are mostly reversible and resolve after the completion or discontinuation of treatment. When toxicity has developed, the potential routes to manage it could be 1) dose reduction of paclitaxel, 2) prolonging the gap between consecutive courses, 3) skipping the next dose, 4) discontinuation of paclitaxel and switching to other agents [6]. Symptomatic relief should be achieved with general measures such as emollients, analgesics, hands or feet elevation, and cold compresses [14]. The role of topical steroids is not very encouraging in PPE and had variable success [6]. Cold compresses and the use of emollients seem to be a more practical approach in relieving symptoms than topical steroids. The role of topical urea-based therapy is also controversial. Systemic steroids and oral pyridoxine can be useful in some cases [6]. We used general measures in our patient and discontinued paclitaxel cycles and switched it to docetaxel, which led to the complete resolution of PPE.

## Conclusions

Paclitaxel can cause PPE. Clinicians should be aware of its occurrence. Its incidence in the literature might be underreported. The use of frozen gloves and frozen socks might prove beneficial to prevent paclitaxel-induced PPE and should be considered in diabetic females. Discontinuation of paclitaxel can reverse the nail and skin toxicity. The decision to continue or discontinue the paclitaxel should be individualized based on patient and disease-related factors. Patients’ perspective in terms of tolerance or acceptance of toxicity and continuation vs discontinuation of paclitaxel should be incorporated in their goals of care. We discontinued paclitaxel in our patient considering the patient’s perspective about her plan of care. 

## References

[1] Crown J, O'Leary M (2000). The taxanes: an update. The Lancet.

[2] Kataria PS, Kendre KP, Patel AA, Tahiliani N, Bhargav V, Parekh H (2018). Rare occurrence of hand-foot syndrome due to paclitaxel: a rare case report. Indian J Pharmacol.

[3] Assi HA, Ayoub ZA, Jaber SM, Sibai HA, El Saghir NS (Breast Care). Management of paclitaxel-induced hand-foot syndrome.

[4] Sibaud V, Lebœuf NR, Roche H (2016). Dermatological adverse events with taxane chemotherapy. Eur J Dermatol.

[5] Miller KK, Gorcey L, McLellan BN (2014). Chemotherapy-induced hand-foot syndrome and nail changes: a review of clinical presentation, etiology, pathogenesis, and management. J Am Acad Dermatol.

[6] Nagore E, Insa A, Sanmartin O (2000). Antineoplastic therapy-induced palmar plantar erythrodysesthesia ('hand-foot') syndrome. incidence, recognition and management. Am J Clin Dermatol.

[7] Gilbar P (2003). Palmar-plantar erythrodysesthesia. J Oncol Pharm Pract.

[8] SERVICES USDOHAH, Institute NC (2017 ). Common Terminology Criteria for Adverse Events (CTCAE). Version 5.0. https://ctep.cancer.gov/protocolDevelopment/electronic_applications/docs/CTCAE_v5_Quick_Reference_8.5x11.pdf.

[9] Biswal SG, Mehta RD (2018). Cutaneous adverse reactions of chemotherapy in cancer patients: a clinicoepidemiological study. Indian J Dermatol.

[10] Poi MJ, Berger M, Lustberg M (2013). Docetaxel-induced skin toxicities in breast cancer patients subsequent to paclitaxel shortage: a case series and literature review. Support Care Cancer.

[11] Engels FK, Verweij J (2005). Docetaxel administration schedule: from fever to tears? a review of randomised studies. Eur J Cancer.

[12] Bardia A, Loprinzi CL, Goetz MP (2006). Hand-foot syndrome after dose-dense adjuvant chemotherapy for breast cancer: a case series. J Clin Oncol.

[13] Naranjo CA, Busto U, Sellers EM (1981). A method for estimating the probability of adverse drug reactions. Clin Pharmacol Ther.

[14] Corazza M, Minghetti S, Borghi A, Virgili A, Ballardini P (2014). Hand-foot syndrome caused by docetaxel with no recurrence after switch to paclitaxel, a different taxane. Int J Dermatol.

[15] Ferreira O, Baudrier T, Mota A, Duarte AF, Azevedo F (2010). Docetaxel-induced acral erythema and nail changes distributed to photoexposed areas. Cutan Ocul Toxicol.

[16] Harris CS, Wang D, Carulli A (2014). Docetaxel-associated palmar-plantar erythrodysesthesia: a case report and review of the literature. J Oncol Pharm Pract.

[17] de Argila D, Dominguez JD, Iglesias L (1996). Taxol-induced acral erythema. Dermatology.

[18] Merimsky O, Inbar MJ (2000). Herceptin-taxol related hand and foot syndrome. Isr Med Assoc J.

[19] Payne JY, Holmes F, Cohen PR, Gagel R, Buzdar A, Dhingra K (1996). Paclitaxel: severe mucocutaneous toxicity in a patient with hyperbilirubinemia. South Med J.

[20] Cruz A, Temu T, Hines-Telang G, Kroumpouzos G (2011). Paclitaxel-induced neutrophilic adverse reaction and acral erythema. Acta Derm Venereol.

